# ^18^F-FDG PET/CT imaging of IgG4-producing MALT lymphoma with multiple site involvement

**DOI:** 10.22038/AOJNMB.2023.73477.1512

**Published:** 2024

**Authors:** Kodai kawaji, Seiji Kurata, Katsuhisa Matsuo, Hiroaki Miyoshi, Jun Akiba, Fumihiko Mouri, Akiko Sumi, Kiminori Fujimoto, Toshi Abe

**Affiliations:** 1 Department of Radiology, Kagoshima University, Graduate School of Medical and Dental Sciences, Sakuragaoka, Kagoshima, Japan; 2 Department of Radiology, Kurume University School of Medicine, Kurume, Japan; 3 Department of Pathology, Kurume University School of Medicine, Kurume, Japan; 4 Department of Diagnostic Pathology, Kurume University Hospital, Kurume, Japan; 5 Division of Hematology and Oncology, Department of Medicine, Kurume University School of Medicine, Japan

**Keywords:** IgG4-producing MALT lymphoma, IgG4-related disease, ^ 18^F-FDG PET/CT, Multiple ^18^F-FDG uptake Plasmacytic differentiation

## Abstract

^18^F-FDG PET/CT is regarded as a modality utilized for the purpose of lesion localization, staging and assessment of treatment response in patients with lymphoma. However, it is difficult that we diagnose among multifocal lymphoma, IgG4-related disease (IgG4-RD), or a combination of both conditions when confronted with multiple sites of ^18^F-FDG uptake with heightened serum IgG4 levels.

We present a case of a 72-year-old male who was suspected of Sjögren’s syndrome based on symptoms of xerostomia accompanied by swelling of the bilateral upper eyelid and salivary glands. Following a diagnostic biopsy that revealed mucosa-associated lymphoid tissue (MALT) lymphoma as a possible finding, ^18^F-FDG PET/CT was conducted, which demonstrated multiple sites of ^18^F-FDG accumulation. While multifocal MALT lymphoma was initially suspected, the coexistence of IgG4-RD could not be definitively ruled out due to the elevated serum IgG4 levels. Subsequent histopathological and immunohistochemical examinations confirmed the diagnosis of IgG4-producing MALT lymphoma. After receiving systemic therapy with rituximab, the swelling of the bilateral upper eyelid and parotid glands resolved upon visual examination, and the serum IgG4 levels returned to within the normal range in a few months. No new lesions were detected during the subsequent follow-up examinations conducted over a period of 3 years.

## Introduction

 MALT lymphoma was first reported by Isaacson and Wright and account for less than 10% of all non-Hodgkin’s lymphomas. Involvement of multiple sites in this disease has been reported in approximately one-third of cases (1). The gastric region is the most commonly affected extranodal site for MALT lymphoma, with the lung, lacrimal and salivary glands being frequent extragastric locations. Other less common sites include the liver, intestine, breast, and thyroid. Thus, the utilization of ^18^F-FDG PET/CT is crucial in providing comprehensive information about the distribution of lesions.

 Here, we report the ^18^F-FDG PET/CT findings of a male patient diagnosed with multifocal MALT lymphoma accompanied by elevated serum IgG4 levels. In cases with elevated serum IgG4 levels, differential diagnosis includes IgG4-RD, which is challenging to distinguish based solely on ^18^F-FDG PET/CT findings. However, based on histopathological and immuno-histochemical observations, we finally confirmed the diagnosis of IgG4-producing MALT lymphoma. Therefore, we conducted a detailed analysis of the ^18^F-FDG PET/CT findings in IgG4-producing MALT lymphoma, incorporating insights from other relevant literature to provide a comprehensive assessment.

## Case Report

 A 72-year-old male presented with clinical suspicion of Sjögren’s syndrome due to xerostomia accompanied by bilateral upper eyelid and salivary glands swelling, persisting for approximately eight months. There was no significant family history, medical history, or medication history noted. Laboratory data were as follows: white blood cell count, 7.8×10^3^/μL (reference range, 3.3-8.6); red blood cell count, 4.08×10^6^/μL (4.35-5.55); hemoglobin, 13.6 g/dL (13.7-16.8); lactate dehydrogenase (LDH), 139 U/mL (124-222); serum total protein, 8.8 g/dL (6.6-8.1); soluble interleukin-2 (IL-2) receptor, 5295 U/mL (157-474); C-reactive protein (CRP), 1.30 mg/dL (0.0-0.14); IgG, 3880 mg/dL (861-1747); and IgG4, 3580 mg/mL (480-1050). IgG4 subclass accounted for 92% of the total IgG fractions (normal, less than 6%). 

 According to the revised diagnostic criteria for Sjögren's syndrome in Japan, which were updated in 1999 and continue to be in use (2), evidence of lymphocytic infiltration was observed in the labial salivary gland tissue. 

 Although salivary gland scintigraphy suggested mild reduction, the Saxon test yielded negative results, and both anti-SS-A and anti-SS-B antibodies were negative as well. Dry eye symptoms were not particularly prominent, and the Schirmer test was not conducted. Therefore, a definitive diagnosis of Sjögren's syndrome was not achieved. The patient underwent a lip biopsy and subsequent excisional biopsy of the left submandibular grand, both of which confirmed the presence of MALT lymphoma.

**Figure 1:**
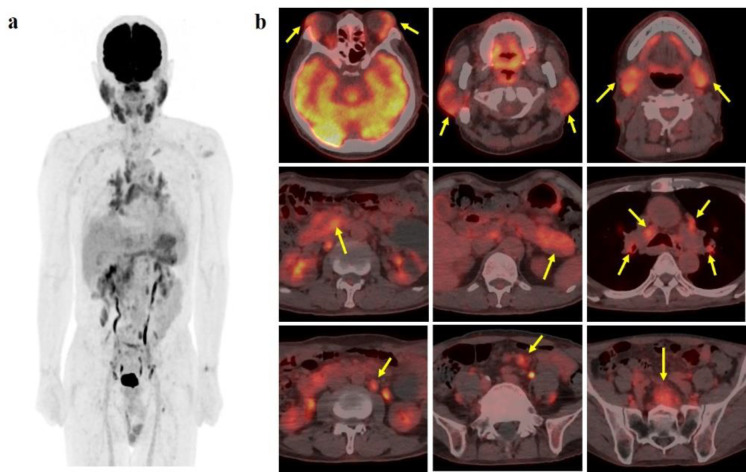
^18^F-FDG PET/CT imaging. a. Maximum intensity projection (MIP) and b. axial fused PET/CT images showed abnormal ^18^F-FDG uptake in multiple sites (**arrows**) including the bilateral lacrimal and salivary glands; duodenum; pancreas; retroperitoneum; lymph nodes (mediastinal, bilateral bronchopulmonary, abdominal para-aorta and mesentery)

 To assess the systemic extent of the disease, ^18^F-FDG PET/CT (GE Discovery Molecular Insights: DMI, GE Healthcare, Waukesha, WI, USA) was conducted. The imaging revealed abnormal ^18^F-FDG uptake was observed in various locations, including the bilateral lacrimal and salivary glands, duodenum, pancreas, retroperitoneum and multiple lymph nodes (mediastinal, bilateral broncho-pulmonary, abdominal para-aorta, and mesentery) (Figures 1a, b-arrows). The maximum standardized uptake value (SUV_max_) of affected lesions ranged from 2.5 to 6.8.

 Elevated levels of serum IgG4 was also observed, indicating that aforementioned multiple lesions with increased ^18^F-FDG uptake could potentially represent not only MALT lymphomas but also IgG4-RD. Based on the 2020 revised comprehensive diagnostic criteria in Japan, pertaining to IgG4-related diseases (3), we have identified manifestations of multiple organ lesions and elevated IgG4 serum levels. Although histologically recognizing the infiltration of IgG4-positive plasma cells, fibrosis was scarcely discernible. Consequently, it falls within the realm of suspected cases; however, it met the criteria for excluding the main condition as MALT lymphoma.

**Figure 2:**
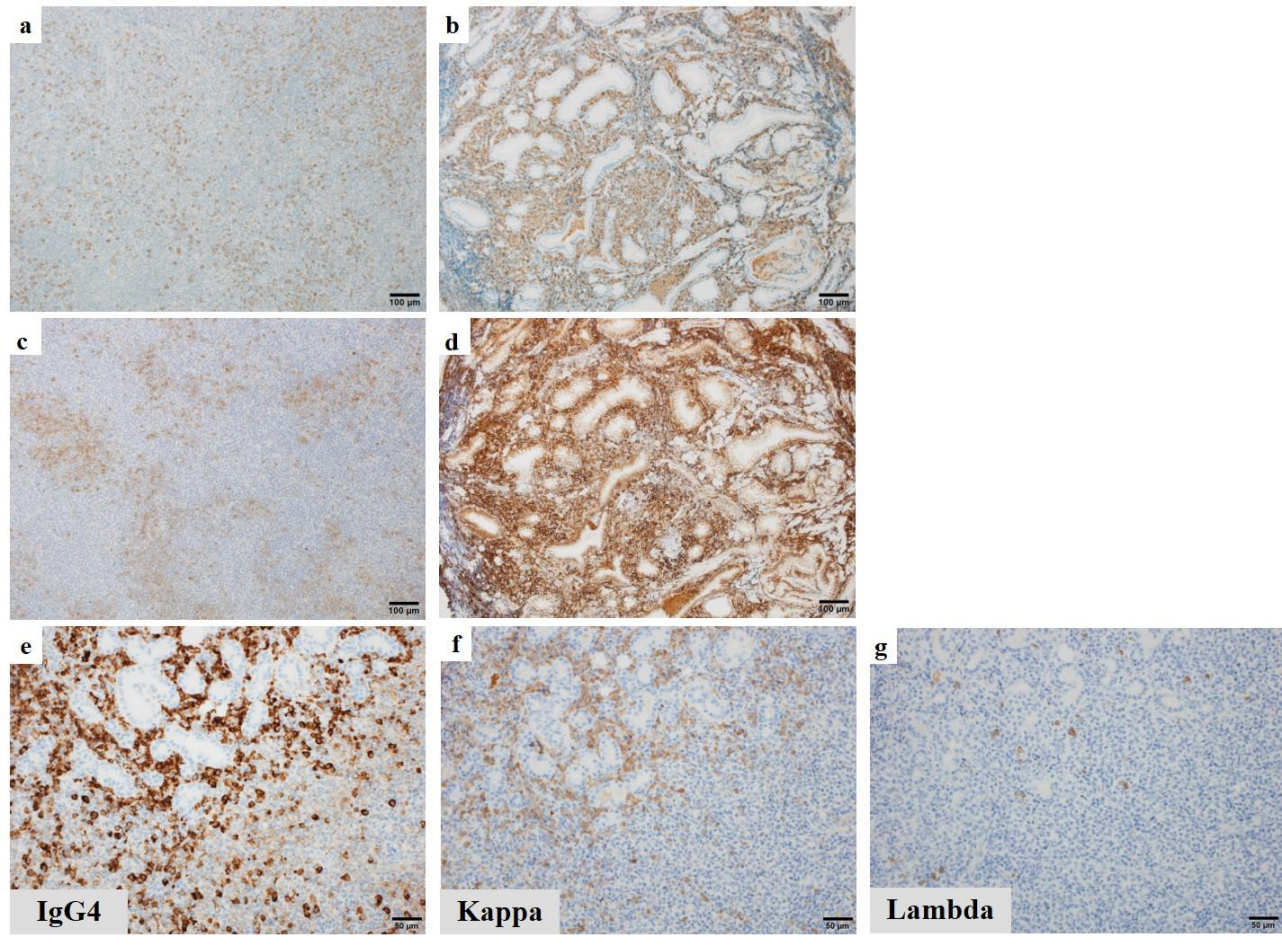
Immunohistochemical features. **a**, **b**. numerous lymphoid cells expressed IgG4 in the left submandibular gland and duodenum (IgG4 staining, 100×). Bar, 100 μm. **c**, **d**. plasmacytic cells were CD138-focally positive in the left submandibular gland and duodenum (CD138 staining, 100×). Bar, 100 μm. **e**, **f**, **g**. IgG4-positive lymphoid cells in the left submandibular gland showed immunoglobulin kappa-light chain monotype restriction (IgG4, kappa-light chain and lambda-light chain staining, 200×). Bar, 50 μm

 Subsequently, an additional biopsy was conducted on the duodenum, which confirmed the presence of MALT lymphoma in the lesions. In both the left submandibular gland and duodenum, numerous lymphoid cells exhibited positive staining for IgG4 (Figures 2a, b), and plasmacytic cells showed focal positively for CD138 (Figures 2c, d). Moreover, the IgG4-positive lymphoid cells in the left submandibular gland displayed immuno-globulin kappa-light chain monotype restriction (Figures 2e, f, g) and a monoclonal rearrangement of the immunoglobulin heavy chain gene was also demonstrated.

 MALT lymphoma, subtype of B-cell lymphoma, necessitates the confirmation of the clonality in both light and heavy chains for its diagnosis. In our case, the co-expression of IgG4 and kappa-light chain in the same cells ultimately led to the identification of an array of IgG4-producing MALT lymphoma. Based on this diagnosis, we administered Rituximab 375 mg/m^2^ once every two weeks for six times. 

**Figure 3:**
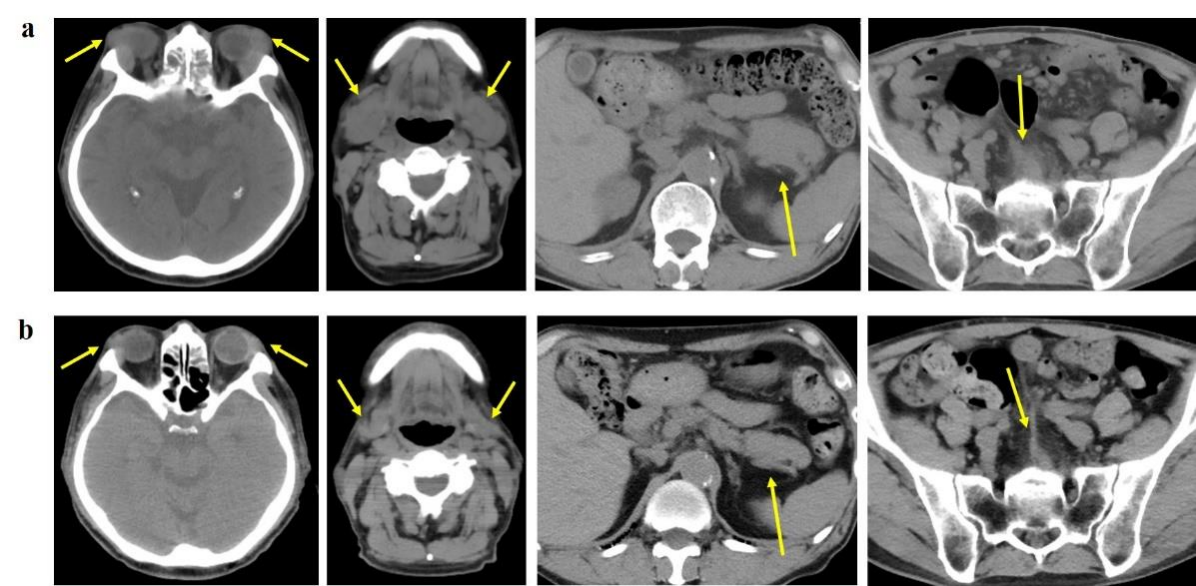
Plain CT imaging. **a**. Prior to systemic therapy, the bilateral lacrimal glands, bilateral submandibular glands, pancreas and retroperitoneum exhibit enlargement (**arrows**). **b**. Following systemic therapy, these lesions demonstrate a tendency towards amelioration of swelling at multiple sites (**arrows**)

 After this systemic therapy, the xerostomia symptoms improved, and visual examination revealed the resolution of swelling in the bilateral upper eyelid and parotid glands. Whole-body plain CT scan demonstrated an improvement in swelling across multiple sites (Figures 3a, b). The serum IgG4 levels decreased to within the normal range in a few months. ^18^F-FDG PET/CT is valuable for assessing the therapeutic efficacy of MALT lymphoma. However, in this case, the determination was based on the clinical symptoms and CT scan evaluation at the discretion of the attending physician. No new lesions were detected during the 3-year follow-up examinations after the therapy.

## Discussion

 MALT lymphomas represent a cohort of indolent B-cell lymphoma that can manifest throughout the body. The foremost organ affected by MALT lymphoma is the stomach, where a definitive correlation has been established between MALT lymphoma and chronic gastritis induced by Helicobactor pylori. Apart from infectious etiologies, chronic inflammation arising from autoimmune conditions like Sjögren’s syndrome or Hashimoto thyroiditis poses a substantial risk for MALT lymphoma development (4).

 In our case involving patients demonstrating elevated serum IgG4 levels, ^18^F-FDG PET/CT unveiled lesions involving multiple organs. IgG4 has been closely associated with autoimmune disease. We concluded that these sites showed a series of IgG4-producing MALT lymphoma based on ^18^F-FDG PET/CT, histopathological and immunohistochemical findings.

 IgG4-producing MALT lymphoma typically originates from the solitarily organ, such as the lacrimal gland, renal hilum and retro-peritoneum, as reported in a few previous studies (5, 6). To the best our knowledge, this is the first documented case of IgG4-producing MALT lymphoma with multiple site involvement detected by ^18^F-FDG PET/CT. 

 MALT lymphoma is known to occur various organs such as the stomach, lung, lacrimal and salivary glands, liver, intestines, breast, and thyroid (1, 7). IgG4-RD also involved diverse organs and tissues, such as the pancreas, pancreatobiliary tract, lacrimal and salivary glands, lung, retroperitoneum and kidney (8, 9). 

 Although previous reports have described various patterns of extragastric MALT lymphoma, there are very few reports involving the duodenum and pancreas, with most of them being single case reports. Multiple MALT lymphomas, including intestinal tract lesions, accounted for less than 5% of all cases (1). 

 Therefore, it might be difficult to differentiate between IgG4-producing MALT lymphoma from MALT lymphoma or IgG4-RD solely based on the ^18^F-FDG PET/CT findings, as in our case. When MALT lymphoma with multiple ^18^F-FDG uptake is accompanied by elevated serum IgG4 levels, IgG4-producing MALT lymphoma should also be considered as the differential diagnosis.

 The demonstration of monoclonality in the left submandibular gland was achieved by subjecting DNA extracted from formalin-fixed paraffin-embedded specimens to electro-phoresis, using primers designed for the IgH (immunoglobulin heavy chain) gene. This resulted in the appearance of a single band, indicating a singular genetic strand. 

 Histopathological characteristics of IgG4-RD typically exhibit infiltration of polyclonal IgG4-expressing plasma cells. The identification of immunoglobulin light-chain restriction and monoclonal rearrangement of the IgH gene may offer potential means of, distinguishing between IgG4-producing MALT lymphoma and IgG4-RD (5).

^18^F-FDG PET/CT provides extensive information concerning the distribution of the disease. In the previous IRRC, ^18^F-FDG PET/CT coverage was limited to Hodgkin lymphoma and diffuse large B-cell lymphoma. However, in 2014, its coverage was broaded to encompass numerous tissue subtypes, including ^18^F-FDG-avid lymphomas. The degree of ^18^F-FDG avidity in MALT lymphoma ranges from 54% to 82% (10), and this variability is contingent upon various factors such as the primary location, tumor size, morphological characteristics (7). 

 Regarding the standardized uptake value (SUV), Qi et al. reported that median SUV was 6.0 (range, 0.7–28.0) in MALT lymphoma (11) and Shimokihara et al. reported that the SUV_max_ was 9.7 in IgG4-producing MALT lymphoma of the renal hilum (6). Thus, it appears that IgG4-producing tissues may exhibit a propensity for greater accumulation in MALT lymphoma, although this was not in our case. Further investigation is warranted to shed more light on this matter.

 In the case of MALT lymphoma, the ability of marginal zone B cells to differentiate into plasma cells is referred to as plasmacytic differentiation (PD). In our case, histo-pathological features, such as the expression of CD138 and the morphology of cell in the left submandibular gland and duodenum, revealed a PD-like phenomenon. IgG4-positive plasmacytic cells exhibited immunoglobulin light-chain restriction in the same lesion. Therefore, our case was considered a potential intense of IgG4-producing MALT lymphoma with PD. We were unable to find any literatures on this phenomenon specifically in relation to ^18^F-FDG PET/CT studies. This phenomenon is commonly observed, particularly in extra-gastric MALT lymphoma, and occurs in up to 30% of all MALT lymphoma cases (12). 

 Rituximab monotherapy may be ineffective in patients with MALT lymphoma with PD whose lymphoma cells do not express CD20. In our case, it might be effective due to the focal expression of CD138. While the disseminated pattern has been reported in extragastric MALT lymphomas (1), not all affected sites were histopathologically confirmed, and the duodenal samples were insufficient to prove monoclonality. It is important to acknowledge this limitation in this report.

## Conflict of interest

 The authors declare that they have no conflict of interest.

## Ethical approval and consent to participate

 All procedures performed in this study were conducted in accordance with the ethical standards of the institutional and/or national research committee and with the 1964 Helsinki declaration and its later amendments or comparable ethical standards. Informed consent was obtained from the participant included in this study.
